# Natural variations in the promoter of *OsSWEET13* and *OsSWEET14* expand the range of resistance against *Xanthomonas oryzae pv*. *oryzae*

**DOI:** 10.1371/journal.pone.0203711

**Published:** 2018-09-13

**Authors:** Abha Zaka, Genelou Grande, Thea Coronejo, Ian Lorenzo Quibod, Chun-Wei Chen, Su-Jein Chang, Boris Szurek, Muhammad Arif, Casiana Vera Cruz, Ricardo Oliva

**Affiliations:** 1 Agriculture Biotechnology Division, National Institute for Biotechnology and Genetic Engineering (NIBGE), Jhang road, Faisalabad, Punjab, Pakistan; 2 Department of Biological Sciences, Pakistan Institute of Engineering and Applied Sciences (PIEAS), P.O. Nilore, Islamabad, Punjab, Pakistan; 3 Rice Breeding Platform, International Rice Research Institute, Metro Manila, Philippines; 4 Taiwan Agricultural Research Institute, Agricultural Research and Extension Station, Council of Agriculture, Guannan, Miaoli District, Taiwan; 5 IRD, CIRAD, Université Montpellier, IPME, Montpellier, France; ICAR-Indian Institute of Rice Research, INDIA

## Abstract

Bacterial blight, caused by *Xanthomonas oryzae* pv. *oryzae* (*Xoo*), is one of the major diseases that impact rice production in Asia. The bacteria use transcription activator-like effectors (TALEs) to hijack the host transcription machinery and activate key susceptibility (*S*) genes, specifically members of the SWEET sucrose uniporters through the recognition of effector-binding element (EBEs) in the promoter regions. However, natural variations in the EBEs that alter the binding affinity of TALEs usually prevent sufficient induction of SWEET genes, leading to resistance phenotypes. In this study, we identified candidate resistance alleles by mining a rice diversity panel for mutations in the promoter of *OsSWEET13* and *OsSWEET14*, which are direct targets of three major TALEs PthXo2, PthXo3 and AvrXa7. We found natural variations at the EBE of both genes, which appeared to have emerged independently in at least three rice subspecies. For *OsSWEET13*, a 2-bp deletion at the 5^th^ and 6^th^ positions of the EBE, and a substitution at the 17^th^ position appear to be sufficient to prevent activation by PthXo2. Similarly, a single nucleotide substitution at position 10 compromised the induction of *OsSWEET14* by AvrXa7. These findings might increase our opportunities to reduce pathogen virulence by preventing the induction of SWEET transporters. Pyramiding variants along with other resistance genes may provide durable and broad-spectrum resistance to the disease.

## Introduction

Rice provides more than 20% of the total caloric intake for half of the world’s population [1]. Under irrigated environments, bacterial blight (BB) represents one of the major biotic constraints to sustain rice production. The disease can easily spread across large cultivated areas causing up to 30% of yield losses [2]. Up to now, the most effective way to control disease epidemics in the field is the introgression of genetic resistance into elite varieties. Development of resistant rice varieties, by pyramiding multiple resistance genes, is a cost-effective disease management strategy as it reduces the net cost for crop production and therefore substantially preserves the yield potential [3].

Disease symptoms are caused by *Xanthomonas oryzae* pv. *oryzae* (*Xoo*), which enters the rice host through wounds or hydathodes and establishes itself in the vascular tissues [4]. For further proliferation in the xylem, *Xoo* relies mainly on type III secretion system-mediated translocation of effector proteins into the host cell. Type III effectors (T3E) mimic host proteins to modulate plant defense response and facilitate access to host nutrients [5, 6]. At least two groups of T3E with a measurable contribution to pathogenesis have been identified. The first group, recognized as *Xanthomonas* outer proteins (Xop), appears to suppress plant innate immunity using diverse biochemical mechanisms [7–9]. The second group includes transcription activator-like effectors (TALEs), which induce the expression of host susceptibility (S) genes to promote a favorable environment for the bacteria [6]. TALE-mediated activation usually occurs by sequence-specific recognition of effector binding elements (EBEs) in the promoter sequence of host target genes [10].

Major TALEs in the global *Xoo* population target at least one of three members of the SWEET sucrose-efflux transporter family [11–14], although, potentially five members of clade III SWEETS can serve as susceptibility genes [12]. The EBE of *OsSWEET11* and *OsSWEET13* are the main targets of Asian *Xoo* populations carrying PthXo1 and PthXo2, respectively [12, 14, 15]. In the same way, *Xoo* also evolved AvrXa7, PthXo3 in Asian [11] and TalC and Tal5 in African populations to target at least three EBEs in the promoter of *OsSWEET14* [12, 16, 17]. This pattern of evolution aligns to the hypothesis that *OsSWEET* activation increases sugar availability within the xylem vessels, providing nutrients during bacterial colonization [18, 19, 12].

Similar to other plants, rice uses multiple mechanisms to defend itself against *Xoo* invasion. A number of resistance genes (*Xa*) evolved to recognize the activity of TALEs and activate defense response. That is the case of *Xa23*, *Xa27*, and *Xa10*, a unique class of decoys activated by cognate TALEs AvrXa23, AvrXa27, AvrXa10 and to trigger cell death [20, 21]. On the other hand, rice blocks the pathogens’ access to sugars as a way to promote *Xoo* starvation and prevent colonization [22]. It has been shown that natural variations disrupting the EBE of *OsSWEET*s generate resistance phenotypes [23]. For instance, PXO99 strain, which carries the PthXo1 effector, is unable to grow on plants carrying the recessive loss-of-susceptibility “resistance” gene *xa13* [6]. A single substitution in the *OsSWEET11* promoter (*xa13*) alters the binding specificity of PthXo1, preventing it from activating the gene [23, 24]. In fact, a number of allelic variants of *OsSWEET11* have been found to be non-responsive to PthXo1 [13, 18, 23].

Lately it was found that TALEs may evolve by codon substitutions and deletion of individual repeats, or reassortment within TALE clusters [25]. These mechanisms are likely to create novel specificities capable of activating unresponsive target variants. Therefore, looking for promoter variants of *OsSWEET* in rice germplasm is of immense importance to expand the level of protection against the pathogen. In the current study, we explored genomic information from more than 3000 rice accessions to identify natural allelic variation on the promoter sequence of *OsSWEET13* and *OsSWEET14* that might expand the level of resistance to *Xoo*. We also tested whether some SNPs/InDel at the relevant EBEs is enough to prevent induction of both *OsSWEET* genes.

## Material methods

### *In silico* mining of a rice diversity panel

For *in silico* mining of *OsSWEET13* and *OsSWEET14*, we analyzed the promoter regions of LOC_Os12g29220 and LOC_Os11g31190, as evidenced in the Nipponbare rice genome annotation project [26]. Using the Rice SNP-seek database [27], we searched for genetic variation in a diversity panel containing 3000 rice genome sequences. Accessions were pre-selected with variations within 1.5kb region upstream of the transcription initiation site of both *OsSWEET* genes. The default setting of the program TALVEZ was used for prediction of the EBE of PthXo1, PthXo2, PthXo3, AvrXa7, TalC, and Tal5 in the promoter of pre-selected accessions [28]. The software uses the repeat variable diresidues (RVD) pattern of each TALE to assess sequence specificity in the rice genome. In the second phase of mining, only accessions with SNP/Indels in the predicted EBE were retained. In addition, selected lines with desirable SNP/Indels from the Pakistani aromatic rice breeding program were also analyzed [29]. The Rice SNP-seek database was used to assess the presence of *Xa4*, *xa5*, *Xa7*, *xa13*, and *Xa21* in selected accessions [30–33].

### Phylogenetic reconstruction

In order to reconstruct the phylogenetic relationships of the selected accessions, three family members (*Os03g37490*, *Os0629950*, and *Os10g20450*) of the multidrug and toxic compound extrusion (MATE) cluster were selected. MATE gene family has been previously reported for explaining different evolutionary trends in *Arabidopsis* and rice [34]. Information regarding start and end position of these MATE genes was obtained from rice genome annotation project [26] and used to extract SNPs from a subset of 366 accessions. An unrooted neighbor-joining tree was constructed with DARwin 6.0.14 software with a bootstrap value of 1000 [35], and the matrix of genetic distances was calculated using the Euclidian distance.

### Plant growth conditions and pathotyping

Seeds of selected accessions (S1 Table) and IR24 (as a susceptible check) were obtained from International Rice Genebank (IRG) at the International Rice Research Institute (IRRI), Philippines and pre-germinated at 37°C for 3 days. Only healthy seedlings were selected and transferred to large pots in the greenhouse. Six reference Philippines *Xoo* strains containing known TALE variants were selected for inoculation i.e. PXO339 carrying PthXo2.1, PXO86 carrying AvrXa7, PXO61 carrying PthXo3, PXO282 carrying PthXo2/AvrXa7, PXO602 carrying PthXo2/AvrXa7.1, and PXO99 carrying PthXo1. Additional strains with unidentified TALEs were also selected, i.e. PXO513, PXO404 and PXO562 (S2 Table). Bacterial inoculum was prepared from three days old pure culture in distilled sterile water with an optical density of 0.2 (OD_600_) and concentration of 1x10^-8^ - 1x10^-9^ CFU/mL. For pathotyping, fully expanded, 3–5 leaves of 21 days and 45 days old plants were clip inoculated [36]. The experiment was replicated three times independently. Data regarding lesion length for each inoculated strain on selected accessions and susceptible check (IR24) was recorded at 14 days post-inoculation (dpi) when lesions were stable. Disease incidence was scored according to IRRI Standard Evaluation System (SES) for rice [37].

### Sequence analysis of predicted EBEs

Three young leaves per accession were harvested, ground in liquid nitrogen and genomic DNA was extracted using the standard CTAB method [38]. DNA was quantified and normalized by ND-1000 NanoDrop spectrophotometer (NanoDrop Technologies; http://www.nanodrop.com) to a concentration of 70ng/μl. Predicted EBEs in Ejali, Khama1183, Super Basmati (SB) and IR24 were amplified using EBE-specific markers (S3 Table). For each PCR reaction, 1.3 μl of DNA, 14.2 μl of UltraPure™ DNase/RNase-free distilled water, 2 μl of 10X buffer, 0.4 μl of 10mM dNTPs, 1 μl of each primer (forward and reverse with a stock concentration of 100 μmol and working concentration of 5 μmol) and 0.1 μl of Taq DNA polymerase was used. Amplification was performed on G-Storm GS1 thermal cycler with an initial denaturation step at 95°C for 5 min, denaturation at 94°C for 30sec, annealing at 94°C for 30sec and extension at 72°C for 1min followed by 34 cycles and ending with a final extension step at 72°C for 10min. PCR amplified products were resolved on 1.2% agarose gel. The amplified product was eluted from the gel and purified using QIAquick Gel Extraction Kit (QIAGEN Sciences, Maryland 20874, USA). Purified products were bidirectionally Sanger sequenced using commercial services and chromatograms were compared to evaluate sequencing quality. Sequenced PCR products were aligned with the reference genome (Nipponbare) using CLUSTALW [39].

### Gene expression analysis

To analyze transcript accumulation of *OsSWEET13* and *OsSWEET14* in response to *Xoo* infection, we infiltrated rice accessions with tester strains containing known TALEs. Plants were grown under controlled environment (28°C and 26°C day and night temperature respectively with a relative humidity of 90%) and artificially inoculated with PXO339 carrying PthXo2.1 and PXO86 carrying AvrXa7. In addition, recombinant PXO99^*TalC*^ (carrying *TalC*) was used to assess the alternative activation of *OsSWEET14* and the empty vector PXO99^EV^ as a control. Twenty-four hours post infiltration (hpi), a total of nine leaves were pooled and stored in liquid nitrogen. Total RNA was extracted using the TRIzol (Invitrogen) method with minor modifications [40].

Quality and concentration of total extracted RNA were measured by 1.65% formaldehyde agarose gel. Five micrograms of RNA from each *Xoo* infiltrated accession was separately treated with amplification-grade DNase1 (Invitrogen) followed by cDNA synthesis using the Super Script® III cDNA synthesis kit (Invitrogen). cDNA derived from 5μg of total RNA was used for each real-time PCR with gene-specific primers (S3 Table). Rice gene *Actin* was used as internal control. The qRT–PCR was performed on Applied Biosystems StepOnePlus RT-PCR system using the PowerUp™ SYBR™ Green Master Mix (Thermo Fisher Scientific). The average threshold cycle (Ct) was used to determine the fold change of mRNA level. The 2ΔΔCt method was used for relative quantification of mRNA accumulation [41].

## Results

### Mining of *OsSWEET13* and *OsSWEET14* promoter polymorphisms in a large database identified potential new sources of resistance

**Fig 1:**
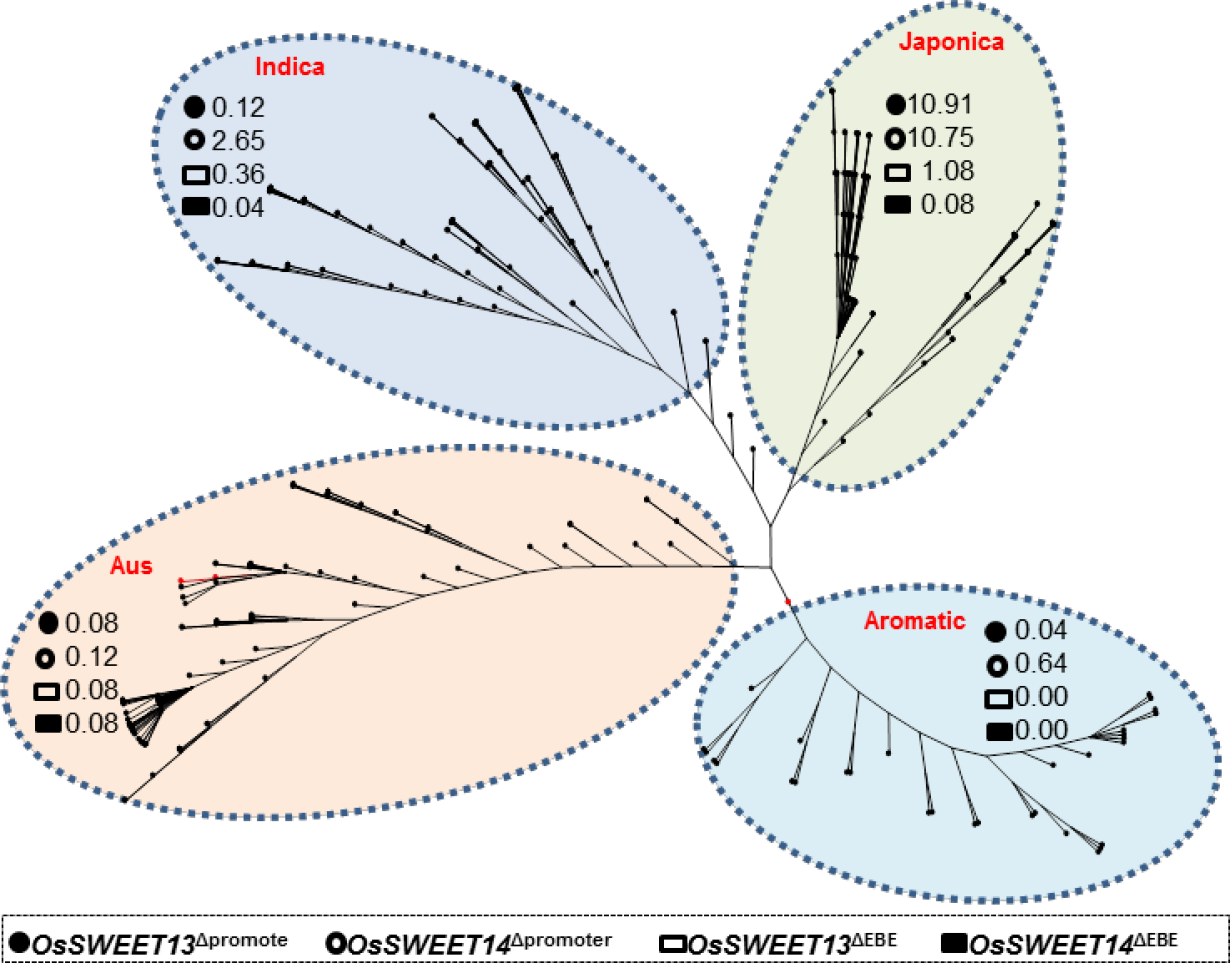
Relative frequency (RF) of mutations (expressed in percentage) in the promoter region of *OsSWEET13* and *OsSWEET14* found in four rice subspecies. The tree topology was constructed using three multidrug and toxic compound extrusion (MATE) genes on a subsample of 366 random accessions. RF of natural variations in the promoter region (circles) and effector-binding element site (squares) of *OsSWEET13* and *OsSWEET14* was calculated based on 3,000 rice accessions. For each rice subspecies, RFs were computed by dividing the number of accessions of that subspecies with natural variations (in promoter region and EBE site) to the total number of accessions in the database and multiplied with 100 to express in percentage. Mutations were more frequently found in japonica, aus and indica subspecies. No aromatic accessions contain EBE specific mutations for both *OsSWEET* genes.

Mining for variations in the promoter sequence of *OsSWEET13* and *OsSWEET14* in the 3k SNP-seek database resulted in 631 accessions with SNPs/InDels within the region 1.5kb upstream of both genes. Based on the phylogenetic tree, different incidence of SNP/InDels was found in the four rice subspecies. Variations in the promoter region from *O*. *sativa* subspecies indica, japonica, aus, and aromatic accessions showed relative frequencies (expressed in percentage) of 10.75, 2.65, 0.12, 0.64 for *OsSWEET13* and 10.91, 0.12, 0.08 and 0.04 for *OsSWEET14*, respectively (Fig 1). Selected accessions from initial mining were further screened to filter out those with SNPs/InDels specifically at the EBE regions. As a result, we selected 41 accessions (S1 Table) with SNPs/InDel variants in the EBE targeted by PthXo2.1, PthXo3, and AvrXa7. The highest relative frequency of EBE mutation for *OsSWEET13* was observed in japonica accessions (1.08) while it was minimum (0.08) in aus accessions. For *OsSWEET14*, the relative frequency of EBE mutation was greater in accessions of japonica subgroup (0.08) and lowest (0.04) in accessions of indica subgroup (Fig 1).

Other than 3K SNP-seek panel, the aromatic germplasm collection [29] was also mined to identify lines with variation in *OsSWEET13* or *OsSWEET14* EBEs. Among the accessions that have breeding value, we found the aromatic variety Super Basmati (SB) to have SNPs/InDels in the *OsSWEET13* EBE which is targeted by PthXo2.1. None of the selected accessions have SNPs/InDels at the predicted EBE targeted by PthXo1 (Part A of S1 Fig). To assess the resistance spectrum of the selected accession we phenotyped all 41 entries against reference strains carrying PthXo2.1, PthXo3, and AvrXa7 (S2 Fig). Thirty-nine of 41 selected accessions showed a decrease in lesions length (LL) ranging from 0.74 cm to 4.75 cm when challenged with PXO339 in comparison with IR24 (susceptible check) i.e. 27.15 cm. Three accessions exhibited reduced lesion length, ranging from 1.9 cm to 5 cm, when challenged with PXO86. Two accessions showed decreased lesion length ranging from 1.5 cm to 3 cm against PXO61 compared with the susceptible check. Interestingly, we found 2 accessions from the aus subgroup, named Ejali and Khama1183, with reduced disease incidence (LL < 5) for all three tested strains (S2 Fig). Hence, Ejali and Khama1183 with pyramided SNPs/InDels in the corresponding EBE of PthXo2.1, PthXo3, and AvrXa7 along with SB were selected for further analysis. As expected, Ejali, Khama1183, and SB were susceptible to PXO99 (Part B of S1 Fig).

### Novel variations identified within *OsSWEET13* and *OsSWEET14* promoter regions

**Fig 2:**
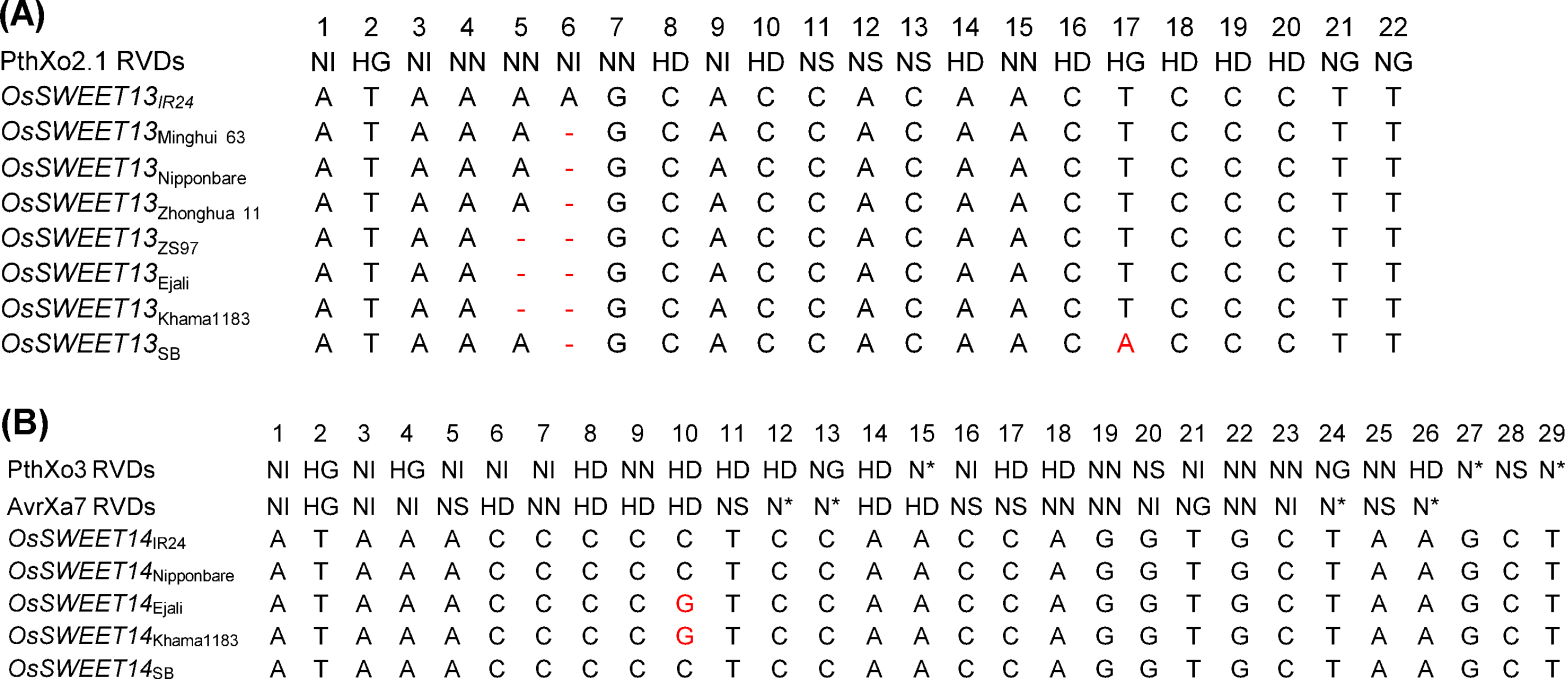
Identification of novel allelic variations in the promoter region of *OsSWEET13* and *OsSWEET14*. (A) Alignment of reported indica, japonica, identified aus and aromatic version of *OsSWEET13* effector-binding element (EBE) showing deletions and substitution at position 5, 6, and 17 targeted by PthXo2.1. (B) Alignment of reported indica, japonica, identified aus and aromatic version of *OsSWEET14* EBE showing a single substitution at position 10 targeted by PthXo3 and AvrXa7. Nucleotide substitutions and deletions are highlighted red, and hyphens, respectively. The repeat variable diresidues (RVD) for PthXo2.1, PthXo3, and AvrXa7 are shown in top of each alignment.

Variations in the EBE of *OsSWEET13* and *OsSWEET14* that affect susceptibility have been described earlier [42, 28]. To validate if the identified SNP/Indels represent novel variants, the regions of the predicted EBE of Ejali, Khama1183, and SB were amplified, sequenced and compared to the reported sequences. Interestingly, novel polymorphisms in the regions targeted by PthXo2, PthXo3 and AvrXa7 were identified (Fig 2). Sequence alignment of predicted EBE in the promoter of *OsSWEET13* in Ejali and Khama1183 with the one in Nipponbare and IR24 confirmed the presence of 2-nucleotide deletion after the putative TATA box region that matches the RVD at position 5 and 6 (Fig 2A). A related deletion was reported by Zhou at al. [14]. Similar to the Nipponbare variant, SB harbors a single nucleotide deletion at position 6 and an additional “T to A” substitution at position 17 (Fig 2A). These natural variations in the EBE of *OsSWEET13* lead to mismatches for three RVDs i.e. NN, NI and HG of PthXo2. For *OsSWEET14*, sequence alignment of predicted EBE in Ejali and Khama1183 validated the presence of a novel single “C” to “G” substitution that matches the RVD at position 10. This single nucleotide substitution in Ejali and Khama1183 generates a gap for RVD “HD” of PthXo3 and AvrXa7. Like IR24, SB did not show any variation in the respective EBE of *OsSWEET14* (Fig 2B).

### Mutations at the EBE appear to prevent activation of *OsSWEET13* and *OsSWEET14*

**Fig 3:**
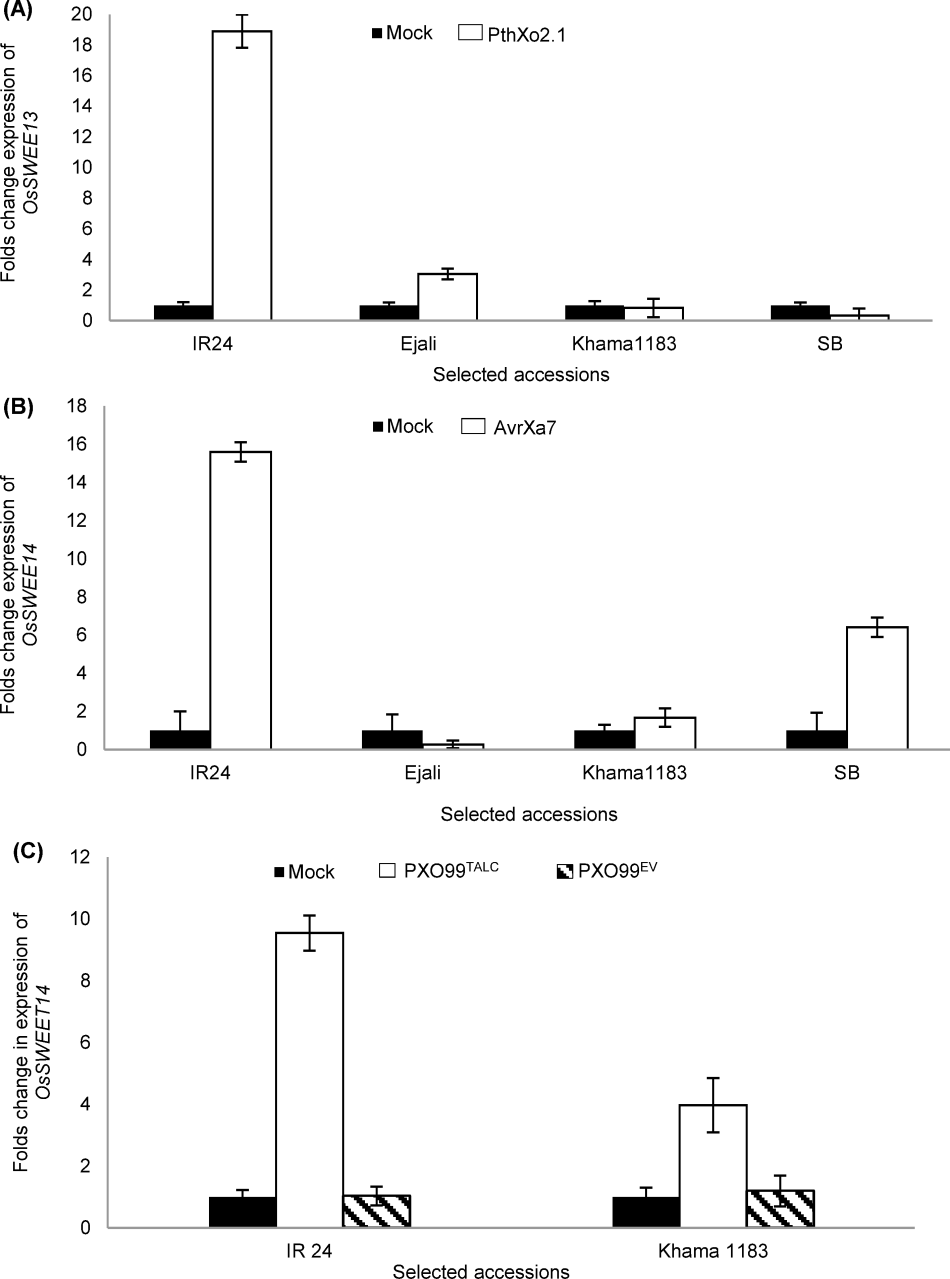
Effector-binding element (EBE) specific mutations prevent the activation of *OsSWEET13* and *OsSWEET14* by *Xanthomonas oryzae* pv. *oryzae* (*Xoo*). Each bar represents average folds change in relative expression at 24 hours post inoculation. Vertical lines on each bar represents ± standard deviation (SD) among three replicates. (A) Transcript accumulation of different version of *OsSWEET13* in mock and PXO339 (carrying PthXo2.1) inoculated plants. (B) Transcript accumulation of different versions of *OsSWEET14* in mock and PXO86 (carrying AvrXa7) inoculated plants. (C) Activation of *OsSWEET14* is possible when a different EBE is used in PXO99^*TalC*^ inoculated plants.

The activation of *OsSWEET13* and *OsSWEET14* by tester *Xoo* strains PXO339, PXO86, and PXO61 have been reported for IR24 [16, 43]. To test if the identified SNPs/Indels in the EBE of *OsSWEET13* and *OsSWEET14* were sufficient to alter the activation of these genes, we inoculated 3-week old plants with tester strains and measured transcript accumulation of both genes after 24 hpi (Fig 3). As expected, PXO339 triggered 18-fold induction of *OsSWEET13* in IR24. Expression of *OsSWEET13* in Khama1183 (0.82-fold) and Ejali (3.12-fold) was restricted (Fig 3A). Although SB features a different type of allelic variation in the EBE, transcript accumulation of *OsSWEET13* in SB was also reduced by 0.22-fold relative to the mock (Fig 3A). In the same way, we observed similar levels of *OsSWEET14* expression in Khama1183 (1.8-fold) and Ejali (0.27-fold) when challenged with PXO86. By contrast, expression of *OsSWEET14* in IR24 (15.5-fold) and SB (6.5-fold) was strongly induced that carry an intact EBE (Fig 3B). To further investigate if *OsSWEET14* is inducible when a different EBE is targeted, we used TalC to activate the mutated version of the gene. We found that *OsSWEET14* can be induced by PXO99^*TalC*^ (3.96-fold) in leaves of Khama1183 but not by PXO99^EV^ or PXO86 (Fig 3C).

### *In silico* and PCR profiling indicate absence of major *Xa* genes that explains the phenotype

To further validate the assumption that reduced disease incidence is associated to impaired expression of *OsSWEET13* and *OsSWEET14* in Khama1183, Ejali, and SB, we checked the presence of *Xa4*, *xa5*, *Xa7*, *xa13*, and *Xa21* using the 3,000-genome information or PCR linked markers. Among them, *Xa7* and *Xa4* appear to be present in Ejali and Khama1183 but none of these genes are known to stop PXO339 or PXO99. In the case of PXO86, which have *AvrXa7*, resistance due to the putative presence of *Xa7* can not be discarded. The recessive *xa5* is an allelic version of the *TFIIA-gamma* transcription factor subunit that reduces the expression of SWEET genes and impairs pathogen colonization [44, 45]. *In silico* and PCR results revealed that Ejali has both alleles of the *TFIIA-gamma* transcription factor subunit in homozygous recessive state (*xa5/xa5*), Khama1183 has heterozygous alleles (*Xa5/xa5*), and SB has both homozygous dominant alleles (*Xa5/Xa5*) (S3 Fig).

### *Xoo* strains with PthXo2 and AvrXa7 variants cannot colonize accessions with EBE-specific mutations

**Fig 4:**
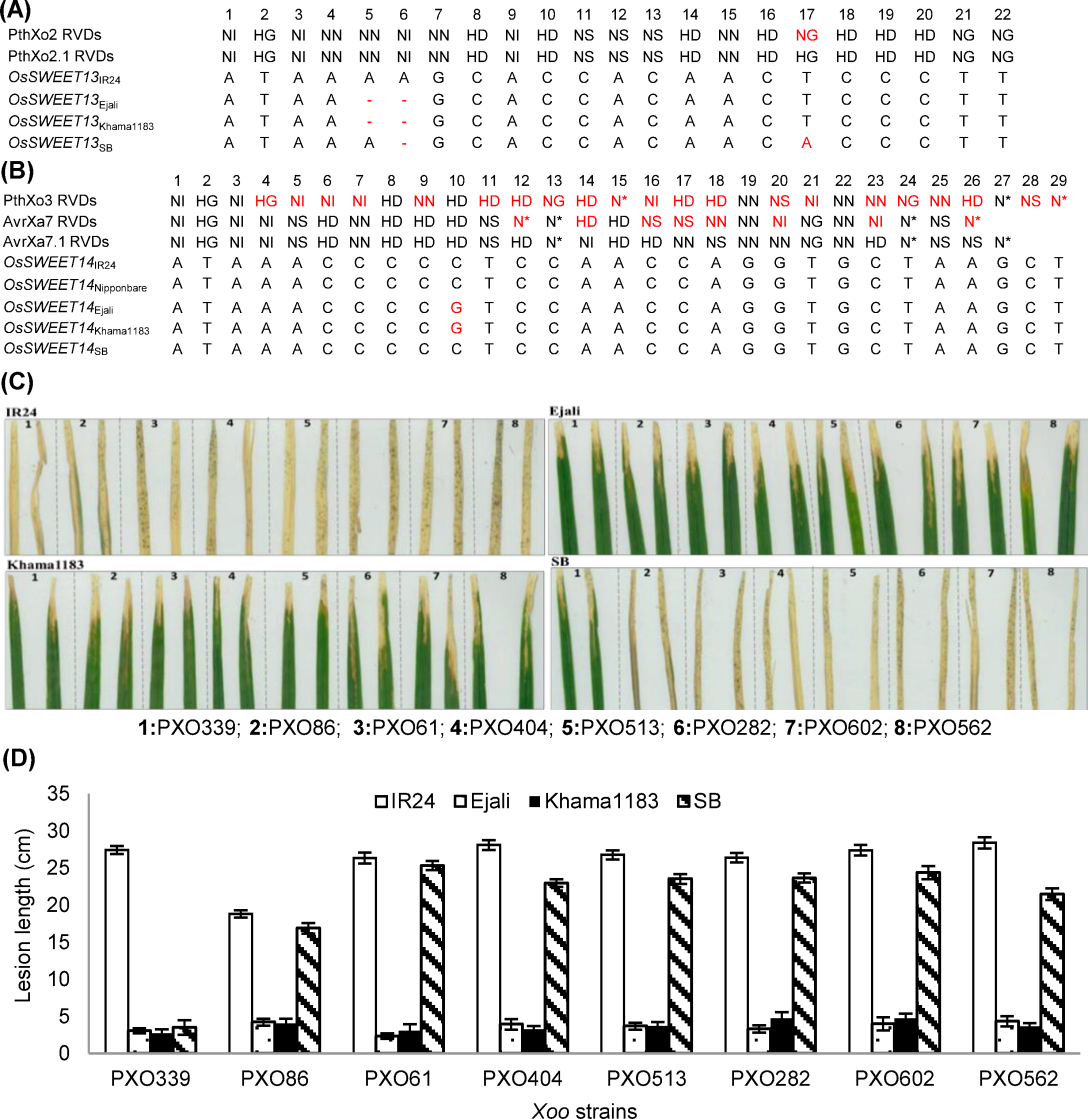
Selected accessions confer broad-spectrum resistance against *Xanthomonas oryzae* pv. *oryzae* (*Xoo*) carrying different variants of PthXo2 and AvrXa7. (A) Alignment of different versions of *OsSWEET13* effector-binding element (EBE) with the repeat variable diresidues (RVDs) of PthXo2 and PthXo2.1. (B) Alignment of different versions of *OsSWEET14* EBE with the RVDs of PthXo3, AvrXa7 and AvrXa7.1. (C, D) Phenotypic response and average lesion length of selected accession inoculated with *Xoo* strains PXO282, PXO602, PXO339 carrying PthXo2 and PthXo2.1, PXO86, PXO282, PXO602 carrying AvrXa7 and AvrXa7.1, PXO61 carrying PthXo3, and PXO404, PXO513, PXO562 carrying unidentified TALe targeting both *OSWEET13* and *OSWEET14*. Accessions were clip inoculated and accessed after 14 days post inoculation. Each bar represents average lesion length of three replicates. Vertical lines on each bar represents ± standard deviation (SD) among three replicates.

To estimate the level of variation among TALEs in relation to the polymorphic sites found in *OsSWEET13* and *OsSWEET14* EBEs, we aligned the RVD sequences of PthXo2 and AvrXa7 with other allelic variants. Both, PthXo2 and PthXo2.1 have similar RVDs except at position 17, where “HG” changes to “NG” (Fig 4A). While these mutations might have distinct binding specificity, we found a naturally existing substitution (“T to A”) at this same particular position in the EBE site of SB *OsSWEET13*. In contrast, AvrXa7 and AvrXa7.1 show significant number of RVDs changes. Interestingly, the substitution at position 10 (“C to G”) aligned to a conserved RVD (“HD”) in AvrXa7, and AvrXa7.1, but also in PthXo3 (Fig 4B).

To further investigate whether *Xoo* strains with allelic variations of PthXo2 and AvrXa7 have potential to colonize the selected accession, we clip-inoculated 21-day old plants with PXO339, PXO86, PXO61, PXO282, and PXO602 representing known TALE variants [46]. In addition we inoculated with PXO513, PXO404, and PXO562 representing a diverse set of virulent strains of *Xoo*. Disease incidence was scored at 14dpi (Fig 4C). Pathotyping results demonstrated that Khama1183 shows broad-spectrum resistance against all tested *Xoo* strains carrying different versions of TALEs. For Khama1183, average lesion length (LL) against tested versions of PthXo2, PthXo3, and AvrXa7 ranged from 2.73 cm to 4.74 cm compared with susceptible IR24 (Fig 4D). We also found that SB was colonized by *Xoo* strain carrying PthXo2 but not by strains carrying PthXo2.1 (Fig 4D).

## Discussion

Plants utilize a number of mechanisms to protect themselves against pathogens. Triggering immunity or disrupting pathogen access to nutrient supply are common strategies that plants evolved to restrict the invading microbes [22]. Understanding how rice limits sugar effluxes will provide a good opportunity to elevate disease resistance against *Xoo*. Since members of the SWEET sucrose-efflux transporter family appear to be hijacked by different *Xoo* populations, pyramiding allelic variants that block *Xoo*-induced sugar release might have an impact on pathogen fitness. In the current study, we accelerated the discovery of rice accessions with naturally mutated EBEs in the promoter sequence of two major susceptibility genes i.e. *OsSWEET13* and *OsSWEET14* by mining a vast diversity dataset. The identified SNP/InDels, which appears to block TALE-mediated activation of *OsSWEET13* and *OsSWEET14* (S4 Table), represent novel variations that appear to have emerged independently in rice subspecies. While Ejali and Khama1183 represent landraces from the aus subgroup, Super Basmati is the only improved variety that renders economic importance. Therefore, stacking *OsSWEETs* variants into SB to systematically control disease epidemics is a likely scenario in breeding programs from exporter countries. We also anticipate that protecting premium Basmati varieties against BB epidemic will effectively reduce yield loses and increase income to farmers.

Our results contribute to the hypothesis that *OsSWEET14* represents a pivotal S-gene in the evolutionary history of *Xoo*. As far as we know, the promoter region contains three overlapping EBE targeted by unrelated TALEs [12]. While Asian and African *Xoo* appear to be genetically different [47], both groups evolved to target the same gene i.e. *OsSWEET14*. For instance, TalC and Tal5 from the African *Xoo* stains BAI3 and MAI1 targets EBEs located upstream and downstream of the overlapping target site of PthXo3 and AvrXa7, from Asian strains PXO61 and PXO86 [17]. Convergent evolution in geographically distant and unrelated pathogens suggests that the activation of *OsSWEET14* was an essential event during *Xoo* adaptation to rice. As predicted by the arm race model, the selection pressure of the pathogen evolves mutation in rice host that prevents *Xoo*-mediated activation of *OsSWEET14*. Interestingly, these mutation events appeared in wild and domesticated rice backgrounds from different geographies, independently. Hutin et al. [42] identified a single 18-bp deletion in the African wild rice *Oryza barthii* that disrupts TalC until Tal5 EBEs. In this work, we report a novel substitution in the EBE of the Asian *Oryza sativa* subspecies. A single change in a cytosine residue (“C” to “G”), appears to be enough to prevent the activation of the gene, and emerged in the target region of Asian effectors PthXo3 and AvrXa7. This pattern of variation aligned with the overall idea that rice was domesticated in different geographies [48] but also that unrelated *Xoo* populations adapt to the same host through a key susceptibility target, independently.

Asian populations of *Xoo* appear to maintain members of the PthXo2 family to mediate the activation of *OsSWEET13* during rice colonization [16, 15]. Similar to other studies [43, 14], our data suggest that mutations in the promoter region of *OsSWEET13* emerged in japonica and indica, independently. So far, the pattern of variation in the promoter of *OsSWEET13* suggests that *Xoo* might be driving the evolution of this gene. PthXo2 is not able to activate the japonica *OsSWEET13* due to a single nucleotide deletion after TATA box region present in resistant varieties i.e. Zhonghua11, Nipponbare, Mudanjing 8, and Minghui 63 [43, 49, 14]. However, the same TALE has a cryptic binding site in the indica promoter of IR24 [14] pointing out to sub species-specific adaptations of PthXo2 members. It is likely that these mutations are maintained in the host populations by balancing selection, where effector diversity could be correlated with S-gene diversity.

So far, two variations at the EBE of *OsSWEET13* have been reported to be susceptible in indica backgrounds i.e. IR24 and Zhenshan97 (ZS97) [50, 51, 14]. Both, *OsSWEET13*_IR24_ and *OsSWEET13*_ZS97,_ can be activated by inoculations with PthXo2.1 and PthXo2-carrying *Xoo* strains [14, 50]. Interestingly, we identified a 2bp-deletion in *OsSWEET13*_Khama1183_ that aligned with the reported sequence of *OsSWEET13*_ZS97_ [14] (Fig 2). However, in our hands, *OsSWEET13*_Ejali_ and *OsSWEET13*_Khama1183_ were not induced when infected with PthXo2 and PthXo2.1. The induction of *OsSWEET13*_ZS97_ may be explained by the presence of heterozygous alleles of *OsSWEET13* in Zhenshan97 and homozygous in Khama1183. Alternatively, Zhenshan97 might have an unknown EBE on *OsSWEET13*, but further evidences are still needed to explain these differences in activation.

Overall, phenotypic assessment of resistance is often challenging due to background effect. The influence of a single nucleotide change might be masked by thousands of other variations in a genotype-specific manner. So at this stage we cannot discard the presence of other resistance genes that might contribute with the phenotype. The particular location of the SNP/InDel within the EBEs, and the lack of *OsSWEET* activation can be presumably correlated with disrupted EBEs in the promoter regions of *OsSWEET13* and *OsSWEET14*. A further genetic analysis is underway and will validate the contribution of these variants to the overall phenotype.

Blocking *Xoo* access to nutrient sources in the rice environment is a promising strategy to gain resistance against BB. However, keeping in mind the genetic diversity of *Xoo* population across different rice growing regions, novel alleles with an expanded range of protection are necessary. Using this approach, we accelerated the discovery of valuable mutations in the rice germplasm. While the direct link between SNP/InDel and phenotype remains to be established for these accessions, our finding provide some hope these EBE variants will be suitable for introgression in elite varieties.

## Conclusions

In modern agriculture, effective control of plant pathogen is one of the core objectives of crop improvement studies. Availability of better knowledge regarding pathogen and host molecular determinants has led to the development of more effective and comprehensive strategies for plant resistance mechanisms. Current study suggest that exploiting natural, loss of function allelic variants of major S genes and their introgression into elite genetic background would diversify the sources of resistance hence providing an additional layer of defense to sustain rice production in BB hotspot region.

## Supporting information

S1 Table List of 41 selected accession with SNP/InDels at the EBE of *OsSWEET13* and *OsSWEET14*. (XLSX)

S2 Table List of *Xoo* strains and their respective TAL effectors used in the study. (DOCX)

S3 Table List of primer for EBE amplification and expression profiling of *OsSWEET13* and *OsSWEET14*. (DOCX)

S4 Table Summary of naturally existing altered EBEs of *OsSWEET13* and *OsSWEET14* in IR24, Ejali, Khama1183 and SB. Activation and lack of activation of both genes in selected accessions by their corresponding TALe is represented with + and–symbol respectively.
(DOCX)

S1 Fig Alignment of PthXo1 RVDs with predicted EBE of *OsSWEET11* and disease incidence of PXO99 on three selected accessions. (A) Alignment of *OsSWEET11* EBE in selected accessions with PthXo1 RVDs contained in PXO99 shows intact EBE without any natural variations. (B) Average lesion length represents susceptible phenotype of selected accessions i.e. Ejali, Khama1183, SB and IR24 inoculated PXO99. Accessions were clip inoculated and accessed for disease incidence 14 days post inoculation. Each bar represents average lesion length of three replicates. Vertical lines on each bar represents ± standard deviation (SD) among three replicates.
(DOCX)

S2 Fig Screening of selected 41 accessions, with SNPs/InDels at predicted EBE of *OsSWEET13* and *OsSWEET14*, against PXO86, PXO61 and PXO339 carrying AvrXa7, PthXo3 and PthXo2.1 respectively. Forty five days old plants were clip inoculated and disease incidence was measured at 14 dpi. Each bar represents average lesion length of three replicates and vertical lines on each bar represents ± standard deviation (SD) among three replicates of each *Xoo* strain. Asterisk indicates accession carrying pyramided SNPs/InDels in the predicted EBE of *OsSWEET13* and *OsSWEET14* with reduced disease incidence for three tested *Xoo* strains.
(DOCX)

S3 Fig Genotyping unveils presence and absence of *xa5* gene in selected accessions. SB, Ejali, Khama1183 and IR24 were genotyped using specific primers (Resistant forward/Susceptible forward + reverse). IRBB5 was used as positive control. Ejali and IRBB5 contain both alleles in homozygous state *xa5/xa5*, SB and IR24 have homozygous dominant (*Xa5/Xa5*) while Khama1183 is heterozygous (*Xa5/xa5*) both alleles.
(DOCX)

## References

[1] Fraiture MA, Roosens NH, Taverniers I, De Loose M, Deforce D, Herman P. Biotech rice: Current developments and future detection challenges in food and feed chain. Trends Food Sci Technol. 2016; 52:66–79.

[2] Mew T. Current status and future prospects of research on bacterial blight of rice. Annu Rev Phytopathol. 1987; 25(1):359–82.

[3] Nalley L, Tsiboe F, Durand-Morat A, Shew A, Thoma G. Economic and Environmental Impact of Rice Blast Pathogen (*Magnaporthe oryzae*) Alleviation in the United States. PLoS One. 2016; 11(12):e0167295 10.1371/journal.pone.0167295 27907101 PMC5131998

[4] NIÑO‐LIU DO, Ronald PC, Bogdanove AJ. *Xanthomonas oryzae* pathovars: model pathogens of a model crop. Mol Plant Pathol. 2006; 7(5):303–24. 10.1111/j.1364-3703.2006.00344.x 20507449

[5] Li T, Huang S, Zhou J, Yang B. Designer TAL effectors induce disease susceptibility and resistance to *Xanthomonas oryzae* pv. *oryzae* in rice. Mol plant. 2013; 6(3):781–9. 10.1093/mp/sst034 23430045

[6] White FF, Yang B. Host and pathogen factors controlling the rice -*Xanthomonas oryzae* interaction. Plant Physiol. 2009; 150(4):1677–86. 10.1104/pp.109.139360 19458115 PMC2719118

[7] Kim JG, Taylor KW, Hotson A, Keegan M, Schmelz EA, Mudgett MB. XopD SUMO protease affects host transcription, promotes pathogen growth, and delays symptom development in *Xanthomonas*-infected tomato leaves. Plant Cell. 2008; 20(7):1915–29. 10.1105/tpc.108.058529 18664616 PMC2518228

[8] Sinha D, Gupta MK, Patel HK, Ranjan A, Sonti RV. Cell wall degrading enzyme induced rice innate immune responses are suppressed by the type 3 secretion system effectors XopN, XopQ, XopX and XopZ of *Xanthomonas oryzae* pv. *oryzae*. PLoS One. 2013; 8(9):e75867 10.1371/journal.pone.0075867 24086651 PMC3784402

[9] Song C, Yang B. Mutagenesis of 18 type III effectors reveals virulence function of XopZ PXO99 in *Xanthomonas oryzae* pv. *oryzae*. Mol Plant Microbe Interact. 2010; 23(7):893–902. 10.1094/MPMI-23-7-0893 20521952

[10] Boch J, Bonas U, Lahaye T. TAL effectors pathogen strategies and plant resistance engineering. New Phytol. 2014; 204(4):823–32. 25539004 10.1111/nph.13015

[11] Antony G, Zhou J, Huang S, Li T, Liu B, White F, et al Rice *xa13* recessive resistance to bacterial blight is defeated by induction of the disease susceptibility gene *Os-11N3*. Plant cell. 2010; 22(11):3864–76. 10.1105/tpc.110.078964 21098734 PMC3015117

[12] Streubel J, Pesce C, Hutin M, Koebnik R, Boch J, Szurek B. Five phylogenetically close rice SWEET genes confer TAL effector mediated susceptibility to *Xanthomonas oryzae* pv. *oryzae*. New Phytol. 2013; 200(3):808–19. 10.1111/nph.12411 23879865

[13] Yang B, Sugio A, White FF. Os8N3 is a host disease susceptibility gene for bacterial blight of rice. Proc. Natl. Acad. Sci. 2006; 103(27):10503–8. 10.1073/pnas.0604088103 16798873 PMC1502487

[14] Zhou J, Peng Z, Long J, Sosso D, Liu B, Eom JS, et al Gene targeting by the TAL effector PthXo2 reveals cryptic resistance gene for bacterial blight of rice. Plant J. 2015; 82(4):632–43. 10.1111/tpj.12838 25824104

[15] Hutin M, Pérez-Quintero AL, Lopez C, Szurek B. MorTAL Kombat: the story of defense against TAL effectors through loss-of-susceptibility. Front Plant Sci. 2015; 6 10.3389/fpls.2015.00006 26347764 PMC4543819

[16] Blanvillain‐Baufumé S, Reschke M, Solé M, Auguy F, Doucoure H, Szurek B, et al Targeted promoter editing for rice resistance to *Xanthomonas oryzae* pv. *oryzae* reveals differential activities for SWEET14‐inducing TAL effectors. Plant Biotechnol J. 2017; 15(3):306–17. 10.1111/pbi.12613 27539813 PMC5316920

[17] Yu Y, Streubel J, Balzergue S, Champion A, Boch J, Koebnik R, et al Colonization of rice leaf blades by an African strain of *Xanthomonas oryzae* pv. *oryzae* depends on a new TAL effector that induces the rice nodulin-3 Os11N3 gene. Mol Plant Microbe Interact. 2011; 24(9):1102–13. 10.1094/MPMI-11-10-0254 21679014

[18] Chen L-Q, Hou B-H, Lalonde S, Takanaga H, Hartung ML, Qu X-Q, et al Sugar transporters for intercellular exchange and nutrition of pathogens. Nature. 2010; 468(7323):527–32. 10.1038/nature09606 21107422 PMC3000469

[19] Chen L-Q, Qu X-Q, Hou B-H, Sosso D, Osorio S, Fernie AR, et al Sucrose efflux mediated by SWEET proteins as a key step for phloem transport. Science. 2012; 335(6065):207–11. 10.1126/science.1213351 22157085

[20] Tian D, Wang J, Zeng X, Gu K, Qiu C, Yang X, et al The rice TAL effector dependent resistance protein *XA10* triggers cell death and calcium depletion in the endoplasmic reticulum. Plant Cell. 2014; 26(1):497–515. 10.1105/tpc.113.119255 24488961 PMC3963592

[21] Zhang J, Yin Z, White F. TAL effectors and the executor R genes. Front Plant Sci. 2015; 6:641 10.3389/fpls.2015.00641 26347759 PMC4542534

[22] Oliva R, Quibod IL. Immunity and starvation: new opportunities to elevate disease resistance in crops. Curr Opin Plant Biol. 2017; 38:84–91. 10.1016/j.pbi.2017.04.020 28505583

[23] Chu Z, Yuan M, Yao J, Ge X, Yuan B, Xu C, et al Promoter mutations of an essential gene for pollen development result in disease resistance in rice. Genes Dev. 2006; 20(10):1250–5. 10.1101/gad.1416306 16648463 PMC1472899

[24] Römer P, Recht S, Strauß T, Elsaesser J, Schornack S, Boch J, et al Promoter elements of rice susceptibility genes are bound and activated by specific TAL effectors from the bacterial blight pathogen, *Xanthomonas oryzae* pv. *oryzae*. New Phytol. 2010; 187(4):1048–57. 10.1111/j.1469-8137.2010.03217.x 20345643

[25] Erkes A, Reschke M, Boch J, Grau J. Evolution of transcription activator-like effectors in *Xanthomonas oryzae*. Genome Biol. Evol. 2017; 1599–1615. 10.1093/gbe/evx108 28637323 PMC5512977

[26] Kawahara Y, de la Bastide M, Hamilton JP, Kanamori H, McCombie WR, Ouyang S, et al Improvement of the *Oryza sativa* Nipponbare reference genome using next generation sequence and optical map data. Rice. 2013; 6(1):4 10.1186/1939-8433-6-4 24280374 PMC5395016

[27] Mansueto L, Fuentes RR, Borja FN, Detras J, Abriol-Santos JM, Chebotarov D, et al Rice SNP-seek database update: new SNPs, indels, and queries. Nucleic Acids Res. 2017; 45(D1):D1075–D81. 10.1093/nar/gkw1135 27899667 PMC5210592

[28] Pérez-Quintero AL, Rodriguez-R LM, Dereeper A, López C, Koebnik R, Szurek B, et al An improved method for TAL effectors DNA-binding sites prediction reveals functional convergence in TAL repertoires of *Xanthomonas oryzae* strains. PLoS one. 2013; 8(7):e68464 10.1371/journal.pone.0068464 23869221 PMC3711819

[29] Bashir K, Khan NM, Rasheed S, Salim M. Indica rice varietal development in Pakistan: an overview. Paddy Water Environ. 2007; 5(2):73–81.

[30] Blair MW, Garris AJ, Iyer AS, Chapman B, Kresovich S, McCouch SR. High resolution genetic mapping and candidate gene identification at the *xa5* locus for bacterial blight resistance in rice (*Oryza sativa* L.). Theor Appl Genet. 2003; 107(1):62–73. 10.1007/s00122-003-1231-2 12677405

[31] Chu Z, Ouyang Y, Zhang J, Yang H, Wang S. Genome-wide analysis of defense-responsive genes in bacterial blight resistance of rice mediated by the recessive R gene *xa13*. Mol Genet Genomics. 2004; 271(1):111–20. 10.1007/s00438-003-0964-6 14730444

[32] Song W-Y, Pi L-Y, Wang G-L, Gardner J, Holsten T, Ronald PC. Evolution of the rice *Xa21* disease resistance gene family. Plant Cell. 1997; 9(8):1279–87. 10.1105/tpc.9.8.1279 9286106 PMC156997

[33] Sun X, Yang Z, Wang S, Zhang Q. Identification of a 47-kb DNA fragment containing *Xa4*, a locus for bacterial blight resistance in rice. Theor Appl Genet. 2003; 106(4):683–7. 10.1007/s00122-002-1117-8 12595998

[34] Wang L, Bei X, Gao J, Li Y, Yan Y, Hu Y. The similar and different evolutionary trends of MATE family occurred between rice and *Arabidopsis thaliana*. BMC Plant Biol. 2016; 16(1):207 10.1186/s12870-016-0895-0 27669820 PMC5037600

[35] Perrier X, Jacquemoud-Collet J. DARwin software. 2006.

[36] Kauffman H. An improved technique for evaluation of resistance of rice varieties to *Xanthomonas oryzae*. Plant Dis Rep. 1973; 57:537–41.

[37] International Network for Genetic Evaluation of R, International Rice Research I. Standard evaluation system for rice. 1996.

[38] Doyle JJ. Isolation of plant DNA from fresh tissue. Focus. 1990; 12:13–5.

[39] McWilliam H, Li W, Uludag M, Squizzato S, Park YM, Buso N, et al Analysis tool web services from the EMBL-EBI. Nucleic Acids Res. 2013; 41(W1):W597–W600. 23671338 10.1093/nar/gkt376 PMC3692137

[40] Chomczynski P, Sacchi N. Single-step method of RNA isolation by acid guanidinium thiocyanate phenol chloroform extraction. Anal. Biochem. 1987; 162(1):156–9. 10.1006/abio.1987.9999 2440339

[41] Livak KJ, Schmittgen TD. Analysis of relative gene expression data using real-time quantitative PCR and the 2− ΔΔCT method. methods. 2001; 25(4):402–8. 10.1006/meth.2001.1262 11846609

[42] Hutin M, Sabot F, Ghesquière A, Koebnik R, Szurek B. A knowledge‐based molecular screen uncovers a broad‐spectrum *OsSWEET14* resistance allele to bacterial blight from wild rice. Plant J. 2015; 84(4):694–703. 10.1111/tpj.13042 26426417

[43] Cheng Q, Mao W, Xie W, Liu Q, Cao J, Yuan M, et al Characterization of a disease susceptibility locus for exploring an efficient way to improve rice resistance against bacterial blight. Sci China Life Sci. 2017; 60(3):298–306. 10.1007/s11427-016-0299-x 28251460

[44] Huang S, Antony G, Li T, Liu B, Obasa K, Yang B, et al The broadly effective recessive resistance gene *xa5* of rice is a virulence effector‐dependent quantitative trait for bacterial blight. Plant J. 2016; 86(2):186–94. 10.1111/tpj.13164 26991395

[45] Yuan M, Ke Y, Huang R, Ma L, Yang Z, Chu Z, et al A host basal transcription factor is a key component for infection of rice by TALE-carrying bacteria. Elife. 2016; 5. 10.7554/eLife.19605 PMC4993585 27472897

[46] Quibod IL, Perez-Quintero A, Booher NJ, Dossa GS, Grande G, Szurek B, et al Effector diversification contributes to *Xanthomonas oryzae* pv. *oryzae* phenotypic adaptation in a semi-isolated environment. Sci Rep. 2016; 6 10.1038/s41598-016-0015-2 27667260 PMC5035989

[47] Soto-Suárez M, Bernal D, González C, Szurek B, Guyot R, Tohme J, et al In planta gene expression analysis of *Xanthomonas oryzae* pathovar *oryzae*, African strain MAI1. BMC Microbiol. 2010; 10(1):170. 20540733 10.1186/1471-2180-10-170 PMC2893596

[48] Hilbert L, Neves EG, Pugliese F, Whitney BS, Shock M, Veasey E, et al Evidence for mid-Holocene rice domestication in the Americas. Nat Ecol Evol. 2017; 1(11):1693 10.1038/s41559-017-0322-4 28993622

[49] Li T, Liu B, Spalding MH, Weeks DP, Yang B. High-efficiency TALEN-based gene editing produces disease-resistant rice. Nat. Biotechnol. 2012; 30(5):390–2. 10.1038/nbt.2199 22565958

[50] Liu Q, Yuan M, Zhou Y, Li X, Xiao J, Wang S. A paralog of the MtN3/saliva family recessively confers race‐specific resistance to *Xanthomonas oryzae* in rice. Plant Cell Environ. 2011; 34(11):1958–69. 10.1111/j.1365-3040.2011.02391.x 21726237

[51] Yuan M, Wang S. Rice MtN3/saliva/SWEET family genes and their homologs in cellular organisms. Mol Plant. 2013; 6(3):665–74. 10.1093/mp/sst035 23430047

