# Supplementary material for: Natural variations in the promoter of OsSWEET13 and OsSWEET14 expand the range of resistance against Xanthomonas oryzae pv. oryzae
Source: PLoS One. 2018 Sep 13;13(9):e0203711. doi: 10.1371/journal.pone.0203711 (PMC6136755; doi:10.1371/journal.pone.0203711)
Supplement: S2 Table — (DOCX) [file pone.0203711.s002.docx]

**S2 Table.** List of *Xoo* strains and their respective TAL effectors used in the study

| **Sr. No** | **Strain name** | **TAL effector** |
| --- | --- | --- |
| 1 | PXO339 | PthXo2.1 |
| 2 | PXO86 | AvrXa7 |
| 3 | PXO61 | PthXo3 |
| 4 | PXO282 | PthXo2 and AvrXa7.1 |
| 5 | PXO602 | PthXo2 and AvrXa7.1 |
| 6 | PXO513 | unidentified major TAL effector targeting *OsSWEET13* and *OsSWEET14* |
| 7 | PXO404 | unidentified major TAL effector targeting *OsSWEET13* and *OsSWEET14* |
| 8 | PXO562 | unidentified major TAL effector targeting *OsSWEET13* and *OsSWEET14* |
