# Supplementary material for: Natural variations in the promoter of OsSWEET13 and OsSWEET14 expand the range of resistance against Xanthomonas oryzae pv. oryzae
Source: PLoS One. 2018 Sep 13;13(9):e0203711. doi: 10.1371/journal.pone.0203711 (PMC6136755; doi:10.1371/journal.pone.0203711)
Supplement: S3 Table — (DOCX) [file pone.0203711.s003.docx]

**S3 Table.** List of primer for EBE amplification and expression profiling of *OsSWEET13* and *OsSWEET14*

| **Sr. No** | **Primer Name** | **Sequence** |
| --- | --- | --- |
| 1 | 11N3-F | GCTCTCACCATTCATTCCACT |
| 2 | 11N3-R | GGGATGCTGAAGAGACATGC |
| 3 | 12N3-F | CCGTATCAGGATTCAGGAATA |
| 4 | 12N3-R | CCAGCCATTTTTGTGTGCTA |
| 5 | cSWEET13-F | GGCAACCTCATATCCTTCAC |
| 6 | cSWEET13-R | TGACGATGTAGATGGTCTCG |
| 7 | cSWEET14-F | CCTAGGCAACATCATCTCCT |
| 8 | cSWEET14-R | CGATGTAGATGGTCTCGATG |
| 9 | *Xa5*/*xa5*-F | Resistant Forward- GCTCGCCATTCAAGTTCTTGAG |
|  |  | Susceptible Forward- GCTCGCCATTCAAGTTCTTGTC |
| 10 | *Xa5*/*xa5*-R | CCTTGATAGAAACCTTGCTCTTGAC |
