# Supplementary material for: Natural variations in the promoter of OsSWEET13 and OsSWEET14 expand the range of resistance against Xanthomonas oryzae pv. oryzae
Source: PLoS One. 2018 Sep 13;13(9):e0203711. doi: 10.1371/journal.pone.0203711 (PMC6136755; doi:10.1371/journal.pone.0203711)
Supplement: S4 Table — Activation and lack of activation of both genes in selected accessions by their corresponding TALe is represented with + and–symbol respectively. (DOCX) [file pone.0203711.s004.docx]

| **Accessions** | ***OsSWEET* target** | **Predicted EBE with natural variations** | **TALe** | **Activation** | **Phenotype** |
| --- | --- | --- | --- | --- | --- |
| IR24 | *OsSWEET13* | ATAAAAGCACCACAACTCCCTT | PthXo2 | + | Susceptible |
| Ejali | *OsSWEET13* | ATAA - - GCACCACAACTCCCTT | PthXo2 | - | Resistant |
| Khama1183 | *OsSWEET13* | ATAA - - GCACCACAACTCCCTT | PthXo2 | - | Resistant |
| SB | *OsSWEET13* | ATAAA **-** GCACCACAACACCCTT | PthXo2 | - | Resistant |
| IR24 | *OsSWEET14* | ATAAACCCCCTCCAACCAGGTGCTAAGCT | AvrXa7 | + | Susceptible |
| Ejali | *OsSWEET14* | ATAAACCCCGTCCAACCAGGTGCTAAGCT | AvrXa7 | - | Resistant |
| Khama1183 | *OsSWEET14* | ATAAACCCCGTCCAACCAGGTGCTAAGCT | AvrXa7 | - | Resistant |
| SB | *OsSWEET14* | ATAAACCCCCTCCAACCAGGTGCTAAGCT | AvrXa7 | + | Susceptible |

**S4 Table.** Summary of naturally existing altered EBEs of *OsSWEET13* and *OsSWEET14* in IR24, Ejali, Khama1183 and SB.
