# Supplementary figures and images for: Natural variations in the promoter of OsSWEET13 and OsSWEET14 expand the range of resistance against Xanthomonas oryzae pv. oryzae
Source: PLoS One. 2018 Sep 13;13(9):e0203711. doi: 10.1371/journal.pone.0203711 (PMC6136755; doi:10.1371/journal.pone.0203711)

**S1 Fig.**


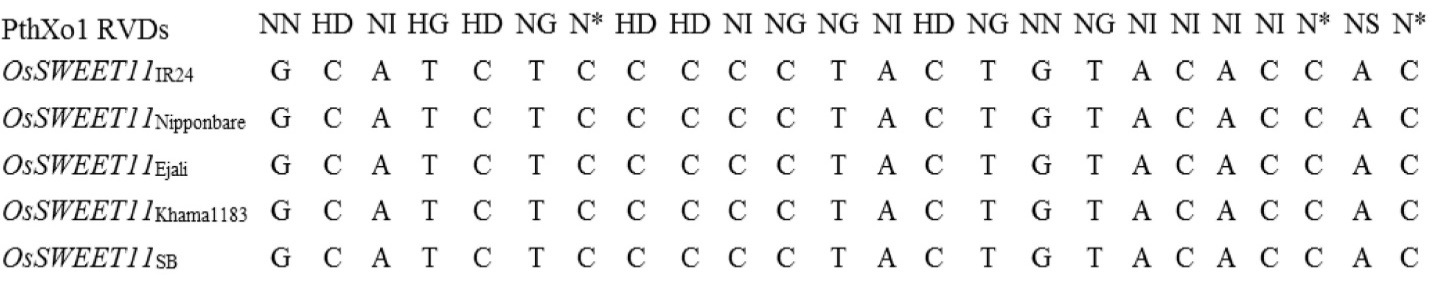

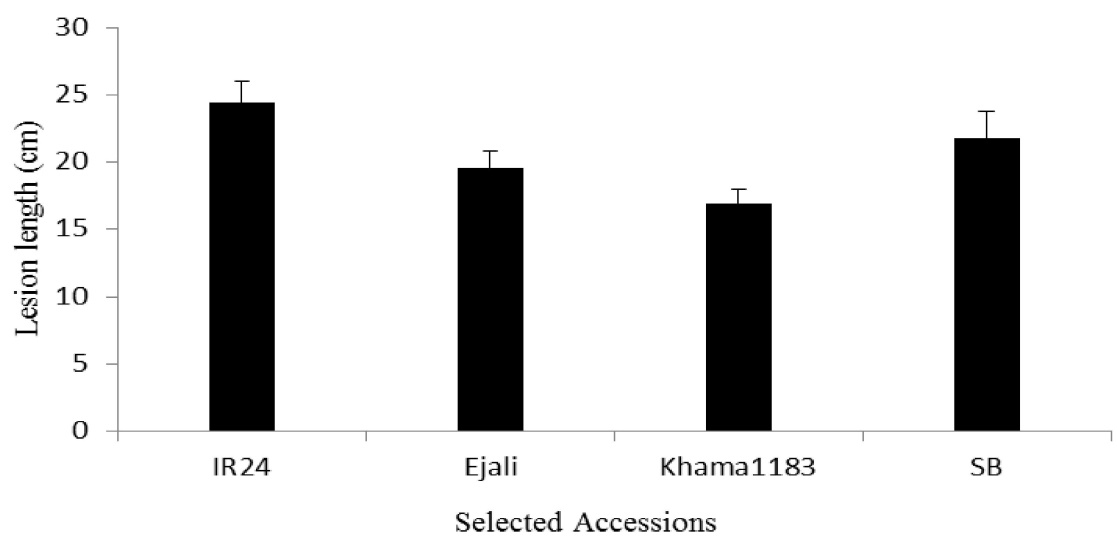


**B)**

**A)**

Supplement: S1 Fig — (A) Alignment of OsSWEET11 EBE in selected accessions with PthXo1 RVDs contained in PXO99 shows intact EBE without any natural variations. (B) Average lesion length represents susceptible phenotype of selected accessions i.e. Ejali, Khama1183, SB and IR24 inoculated PXO99. Accessions were clip inoculated and accessed for disease incidence 14 days post inoculation. Each bar represents average lesion length of three replicates. Vertical lines on each bar represents ± standard deviation (SD) among three replicates. (DOCX) [file pone.0203711.s005.docx]

**S2 Fig.**


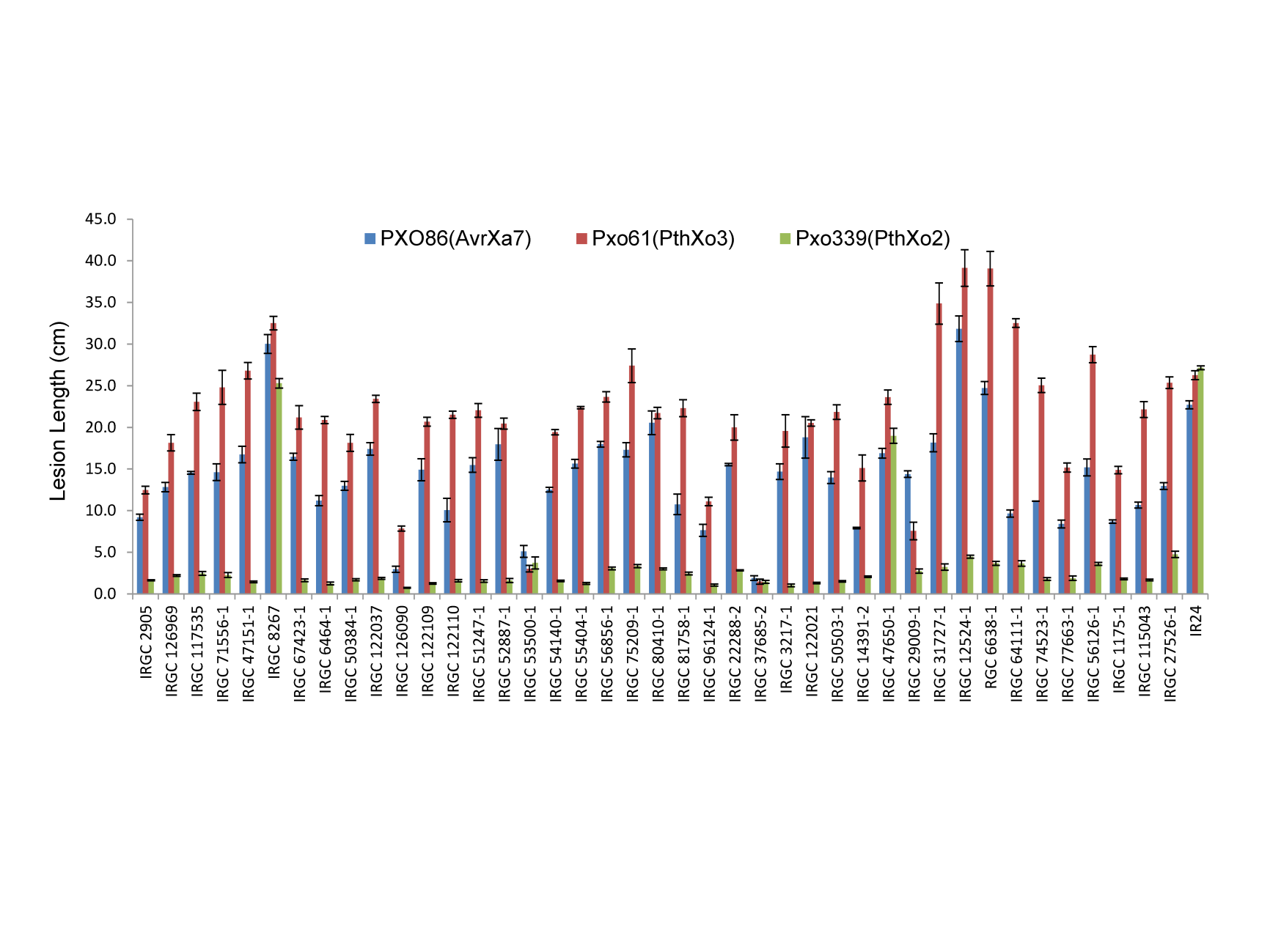

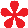

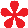

Supplement: S2 Fig — Forty five days old plants were clip inoculated and disease incidence was measured at 14 dpi. Each bar represents average lesion length of three replicates and vertical lines on each bar represents ± standard deviation (SD) among three replicates of each Xoo strain. Asterisk indicates accession carrying pyramided SNPs/InDels in the predicted EBE of OsSWEET13 and OsSWEET14 with reduced disease incidence for three tested Xoo strains. (DOCX) [file pone.0203711.s006.docx]

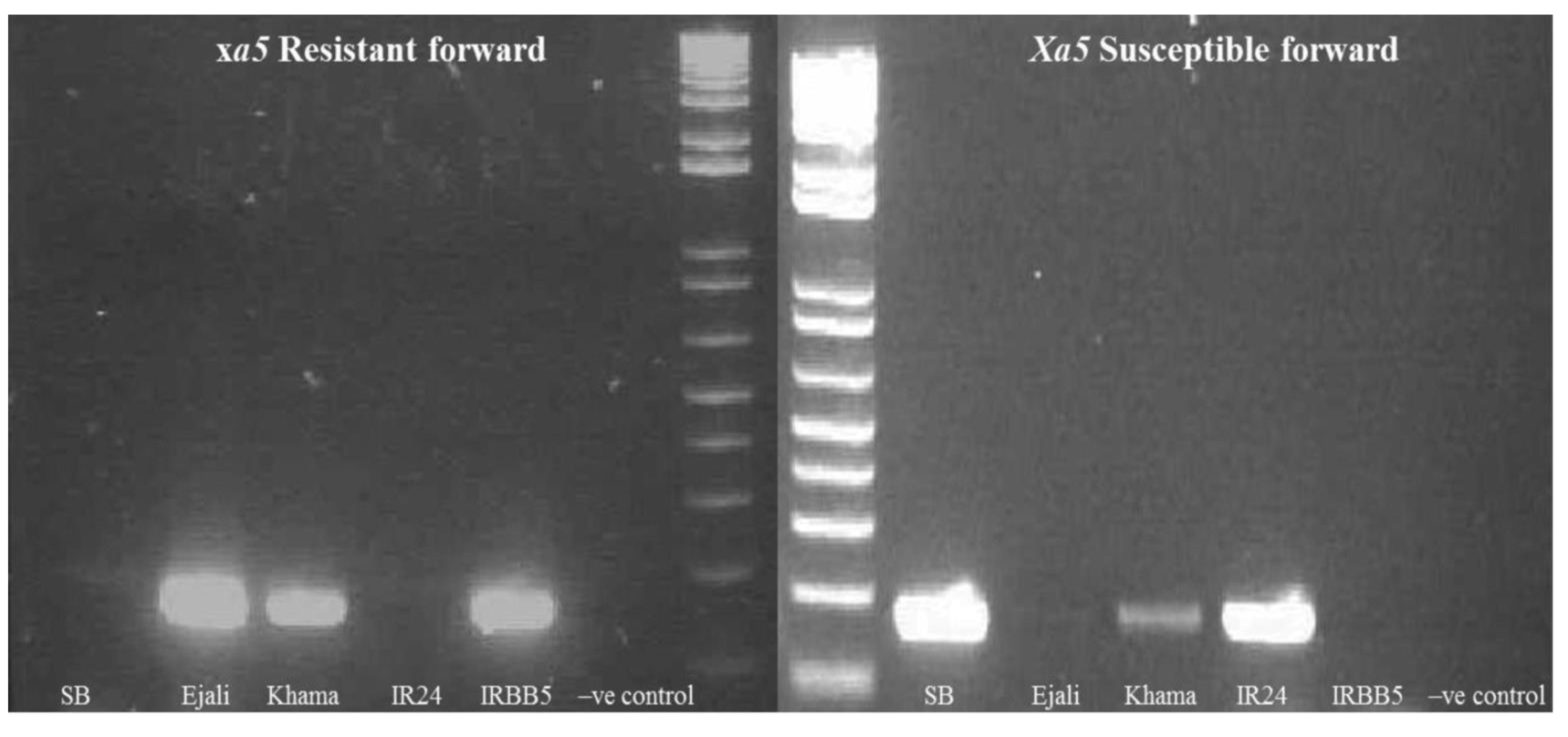
**S3 Fig.**

Supplement: S3 Fig — SB, Ejali, Khama1183 and IR24 were genotyped using specific primers (Resistant forward/Susceptible forward + reverse). IRBB5 was used as positive control. Ejali and IRBB5 contain both alleles in homozygous state xa5/xa5, SB and IR24 have homozygous dominant (Xa5/Xa5) while Khama1183 is heterozygous (Xa5/xa5) both alleles. (DOCX) [file pone.0203711.s007.docx]
